# Christiaan Sybesma (August 31, 1928–January 31, 2018), an extraordinary biophysicist of our time

**DOI:** 10.1007/s11120-020-00734-x

**Published:** 2020-04-02

**Authors:** Wim J. Vredenberg, Govindjee Govindjee

**Affiliations:** 1 Department of Plant Physiology, Wageningen University & Research, Wageningen, The Netherlands; 2 Department of Plant Biology, Department of Biochemistry and Center of Biophysics and Quantitative Biology, University of Illinois at Urbana, Champaign, IL 61801 USA

**Keywords:** P840, FMO (Fenna-Matthews-Olson) protein, Excitation energy transfer, Reaction center BChl a, Biophysics, *Rhodospirillum rubrum*

## Abstract

We provide here a brief Tribute to Christiaan Sybesma (1928–2018), a highly respected biophysicist of our time. We remember him by giving a brief highlight of his life and a glimpse of his outstanding contributions in photosynthesis. He was a charming and highly respected scientist of our time.

Figure 1 shows a photograph of Christiaan Sybesma, in a serene and thoughtful mood, on Christmas 2015 (see Fig. 1).

**Fig. 1:**
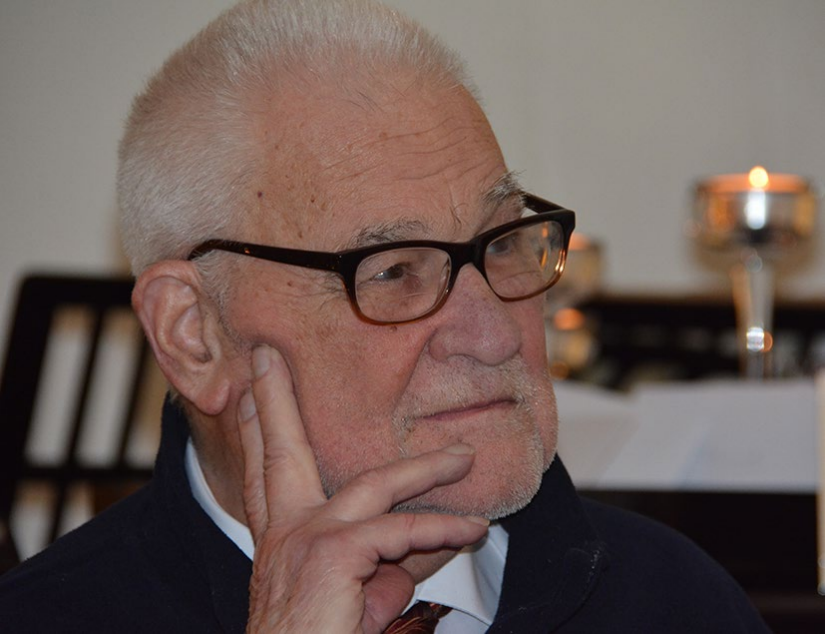
A 2015 photograph of Christiaan Sybesma taken by Chris Sybesma Jr

Christiaan (Chris) Sybesma was born in Bandung, Indonesia (at that time ‘Dutch East Indies, a colony of The Netherlands). He was the eldest son of Feike Sybesma and Cécile Heyman. His father was the Head of the Teachers' Training College, and later served as a Member of the *Raad van Indië*, a parliamentary body representing the Colony. During the Japanese occupation, from 1942, Chris’s father was imprisoned, while the other members of the family, Cécile and the five children, spent 3 years in various Japanese ’concentration camps’. They all survived. In 1945, the family returned to the Netherlands, where family life resumed. Chris finished high school in Amsterdam; in 1948, he began his studies in Physics at the Technical University in Delft. (The Netherlands). There, Chris obtained his PhD in Applied Sciences, with a thesis on ‘*Measurements of continuous energy distributions of gamma rays in a scattering medium’*, under the mentorship of Prof. J.J. Went (Sybesma 1961). Most of the experimental thesis work was done in the Laboratory of Metabolic Diseases and Endocrinology of the Leiden University Medical Center, headed by Prof. A. Querido. From this early work and associated scientific activities, we learn about one of Chris’ strong interests in applying biophysics with the goal of obtaining insight in the inner working of the physiological processes in living systems and in providing means to monitor them.

In 1961, Chris entered the field of plant biophysics when he joined the research group of John M. Olson (1928–2018; see Blankenship et al. 2018) at the Brookhaven National Laboratory, Brookhaven (USA). Here, he focused primarily on the bioenergetic events in green sulfur bacteria. He successfully showed that bacteriochlorophyll (BChl) c, a major green pigment in *Chloropseudomonas ethylicum*, transfers excitation energy to BChl a, a protein (later named as the Fenna-Mathews-Olson (FMO) protein-complex), which, in turn, transfers energy to a reaction center BChl a (P 840) for photochemistry (Sybesma and Olson 1963).

In 1964, Chris joined the Dutch biophysics group of Louis N.M. Duysens (1921–2015; see Govindjee and Pulles 2016) at the University in Leiden. It was a hectic period during which break-through research was being done leading to the discoveries and spectrophotometric identification and functioning of photochemically-active reaction centers (RCs: P700 and P680) in the photosystems I (PSI) and II (PSII) in algae and plants, as well as BChl a-containing RCs in photosynthetic bacteria. Sybesma’s contribution therein has been the identification of P840 serving as the reaction center in green bacteria (Sybesma and Vredenberg 1964).

From 1966 to 1972, Chris served as an Associate Professor of Biophysics (in the Department of Physiology & Biophysics) and Botany (now Plant Biology), at the University of Illinois at Urbana-Champaign (UIUC), where he was a member of the prestigious Photosynthesis group of Eugene Rabinowitch (see Govindjee et al. 2019) and his successor Govindjee (one of the authors) in the Department of Plant Biology at UIUC. Here, he also served as Head (Director) of the Biophysics group. He taught Biophysics, and was a major advisor of PhD students, including Charles Fowler and William Smith. With Fowler, he published novel results on the electron transport reactions in *Rhodospirillum rubrum* (Sybesma and Fowler 1968). Rajni Govindjee joined his research group, as a senior researcher, and worked with him on several topics including pyridine nucleotide reduction, and its relation to electron transport in young and old cells of *Rhodospirillum rubrum* (Govindjee and Sybesma 1970).

From UIUC, Urbana, Illinois, Chris returned to Europe in 1972, and was appointed as Professor of Biophysics in the Physics Department of the Vrije Universiteit Brussel (VUB). This university was newly founded in the early 1970s after the Dutch language Division of the Université libre de Bruxelles (ULB) had become an independent university: the Vrije Universiteit Brussel (VUB). Here he started a photobiophysics group with Luit Slooten as his first research associate; he also worked with Marc Symons and Christine Swysen on carotenoid absorbance changes in *Rhodopseudomomas sphaeroides* (Swysen et al. 1977). Further, he did joint research on the ecological aspects of plant photobiology with research groups of Marc Montagu and David Inzé at the University of Ghent, Belgium (Slooten et al. 1995).

In 1977, Sybesma published his excellent textbook “An Introduction to Biophysics” for both undergraduate and graduate students (Sybesma 1977). This book was used around the world in beginning Biophysics courses; Chris revised and updated it in 1989, and published it as “ Biophysics- an Introduction,” which is still available.

Chris Sybesma was the prime organizer and chairman of the Sixth International Congress on Photosynthesis, held in Brussels, August 1–6, 1983. He considered this congress as the Eighth in a (still proceeding) sequence that started as ‘European’ in 1963 with number -minus 1 in Gif-sur-Yvette, near Paris, France (organizer A. Moyse), and in 1965, followed by number zero in Woudschoten (The Netherlands), organized by Jan Thomas and Joop Goedheer. He edited the ~ 1200 contributions (covering 4 volumes) of the Congress Proceedings ‘Advances in Photosynthesis Research’ (Sybesma 1984).

Figure 2 shows two photographs of Chris Sybesma, reflecting on his being a wonderful host to his friends and visitors.

**Fig. 2:**
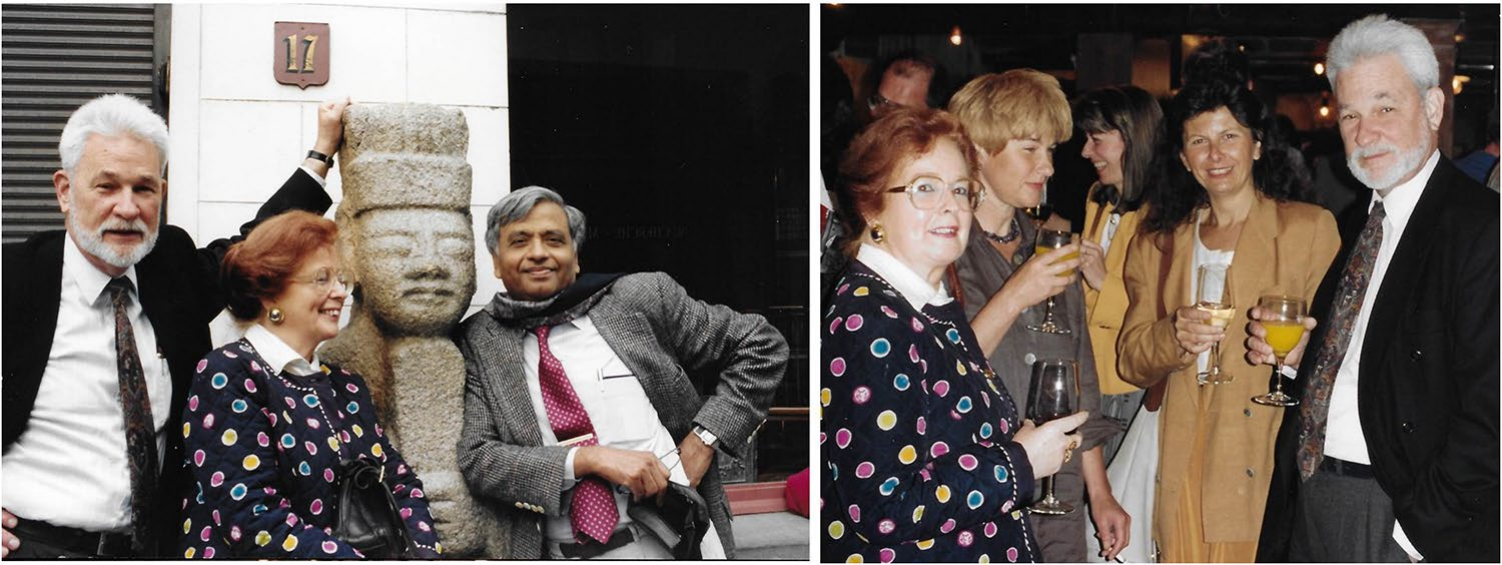
(Left). Left to right: Chris Sybesma, Neri Sybesma and one of us (Govindjee Govindjee). (Right): Neri, Chris's sister Tina Sybesma, soprano Ann Crossley and Chris. These photographs were taken in early 1980s, on the occasion of a concert of Bach cantata’s in the* Eglise des Minimes* in Brussels. Source: Archives of Rajni Govindjee

The academic environment at the VUB/ULB, and in particular, the contacts with Prof. Ilya Prigogine, well-known for his ideas about issues relevant to views and discussions on actual interactions between (theoretical) physics and philosophy of science gave him spiritual inspiration. He was the co-organizer of several international congresses. The one entitled ‘*Einstein meets Margritte: an interdisciplinary reflection on science, nature, human action, and society*’, held in 1995 in Brussels, marked the 25th anniversary of the VUB. He wrote the book (in Dutch) ‘*De werkelijkheid heeft mij niet nodig*’ (Sybesma 2002). This was also the title of his farewell speech he delivered in 1998 at the occasion of his retirement from the VUB. The title, in translation ‘*Reality does not need me*’, is the last line of a poem of the Portuguese poet Fernando Pessoa (see the quote at the top of this Tribute). The last part of this poem was at the top of the official announcement of his death (see https://www.wimvanderlinden.be/rouwbrieven/ChristiaanSybesma.pdf). Chris leaves behind his wife Neri, his two sons Chris and Bessel, three grandchildren, and two great-grandsons. His daughter Barbara had passed away in 2017.

Chris Sybesma has associated not only with Lou Duysens, John Olson, and Eugene Rabinowitch (top authorities, mentioned above), but also with Bessel Kok, discoverer of P700, reaction center of PSI (see Myers 1981). Chris was a highly respected member of the International Community of Photosynthesis Research, populated by a large number of colleagues of his generation and their successors. He deserves to be remembered by the younger and forthcoming generation as a charming and communicative scientist with renowned stature.

We end this Tribute with a 1980s portrait, when he was at VUB, in Belgium, and a 1993 photograph when he was in Bandung, Indonesia, where he taught Biophysics at the Technical University. We see him here as a thinking and a relaxed person: this is how we remember him (Fig. 3).

**Fig. 3:**
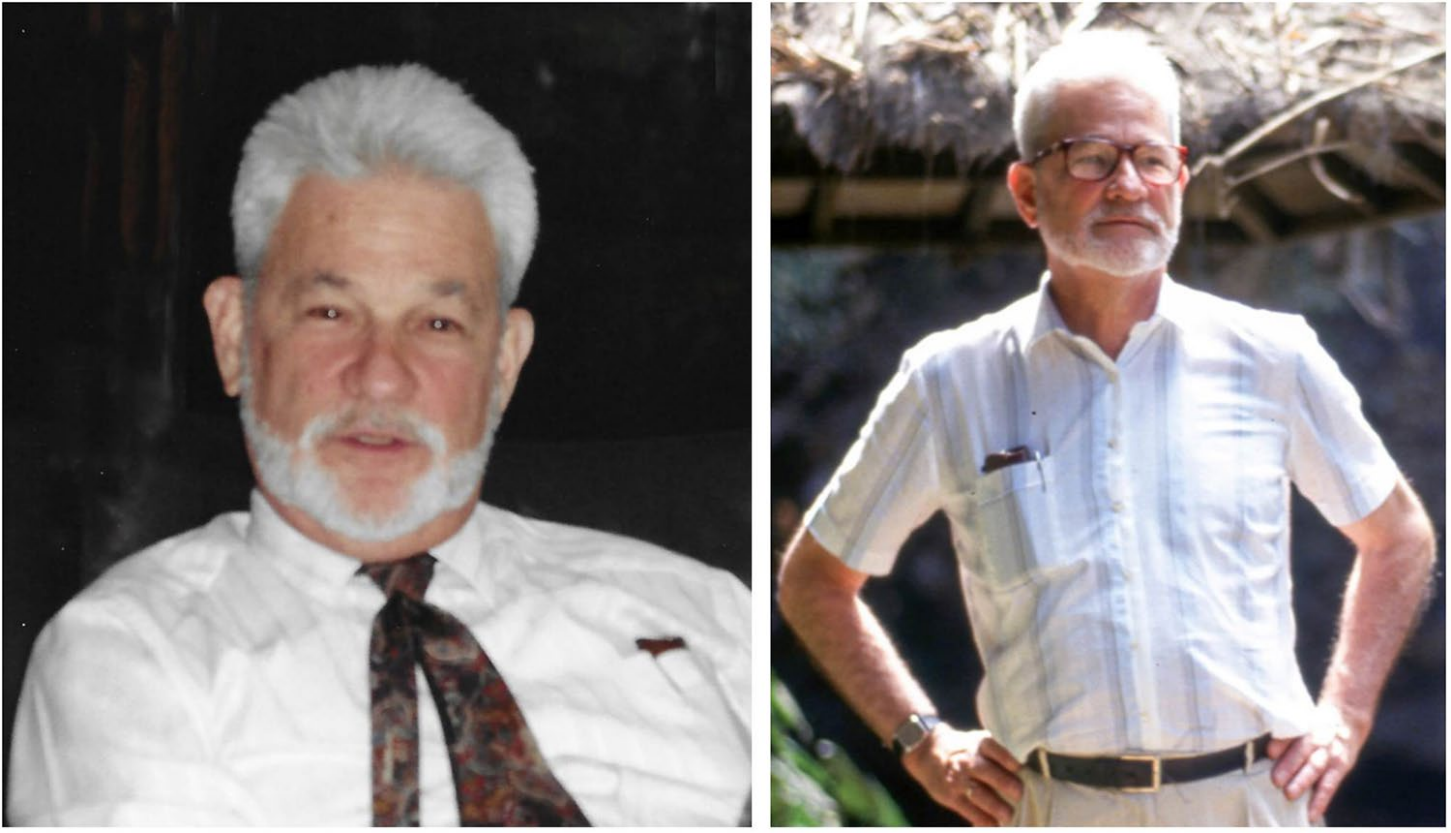
(Left) A 1980s portrait of Chris Sybesma in Belgium, taken by one of us (GG) in a formal setting. Source: Archives of Rajni Govindjee. (Right): A 1993 photograph of Christiaan Sybesma, in an informal setting in Bandung, Indonesia. Source: Sybesma Archives
